# Codevelopment of Microbiota and Innate Immunity and the Risk for Group B Streptococcal Disease

**DOI:** 10.3389/fimmu.2017.01497

**Published:** 2017-11-10

**Authors:** Julia Kolter, Philipp Henneke

**Affiliations:** ^1^Center for Chronic Immunodeficiency (CCI), Medical Center – University of Freiburg, Faculty of Medicine, University of Freiburg, Freiburg, Germany; ^2^Faculty of Biology, University of Freiburg, Freiburg, Germany; ^3^Center for Pediatrics and Adolescent Medicine, Medical Center – University of Freiburg, Freiburg, Germany

**Keywords:** *S. agalactiae*, Group B *Streptococcus*, cellular innate immunity, microbiome, colonization, sepsis

## Abstract

The pathogenesis of neonatal late-onset sepsis (LOD), which manifests between the third day and the third month of life, remains poorly understood. Group B *Streptococcus* (GBS) is the most important cause of LOD in infants without underlying diseases or prematurity and the third most frequent cause of meningitis in the Western world. On the other hand, GBS is a common intestinal colonizer in infants. Accordingly, despite its adaption to the human lower gastrointestinal tract, GBS has retained its potential virulence and its transition from a commensal to a dangerous pathogen is unpredictable in the individual. Several cellular innate immune mechanisms, in particular Toll-like receptors, the inflammasome and the cGAS pathway, are engaged by GBS effectors like nucleic acids. These are likely to impact on the GBS-specific host resistance. Given the long evolution of streptococci as a normal constituent of the human microbiota, the emergence of GBS as the dominant neonatal sepsis cause just about 50 years ago is remarkable. It appears that intensive usage of tetracycline starting in the 1940s has been a selection advantage for the currently dominant GBS clones with superior adhesive and invasive properties. The historical replacement of Group A by Group B streptococci as a leading neonatal pathogen and the higher frequency of other β-hemolytic streptococci in areas with low GBS prevalence suggests the existence of a confined streptococcal niche, where locally competing streptococcal species are subject to environmental and immunological selection pressure. Thus, it seems pivotal to resolve neonatal innate immunity at mucous surfaces and its impact on microbiome composition and quality, i.e., genetic heterogeneity and metabolism, at the microanatomical level. Then, designer pro- and prebiotics, such as attenuated strains of GBS, and oligonucleotide priming of mucosal immunity may unfold their potential and facilitate adaptation of potentially hazardous streptococci as part of a beneficial local microbiome, which is stabilized by mucocutaneous innate immunity.

## Introduction

Neonatal sepsis occurs as two distinct clinical entities either in the first 72 h of life as early-onset disease (EOD), resulting from *in utero* or intrapartum infection, or during the following 3 months as late-onset sepsis (LOD). In both cases, the Gram-positive, β-hemolytic Group B *Streptococcus* (GBS) is one of the most prevalent bacterial species in blood and cerebrospinal fluid. As a consequence, pregnant women undergo routine or targeted screening for GBS in the last third of pregnancy in many Western European countries and the USA. In case of positive testing, women receive preventive intrapartum antibiotics during delivery (1). Since approximately 20–30% of all pregnant women are colonized, this prevention strategy affects an estimated 1 million women every year in the US alone. In other countries such as the Netherlands, a risk-based approach has been adopted, i.e., antibiotics are only administered in case of additional risk factors such as premature labor, intrapartum fever, bacteriuria, prolonged membrane rupture or previous children with GBS disease.

Before the use of antibiotic prophylaxis, the GBS sepsis incidence exceeded 1 in 1,000 children with high case fatality rates (2, 3). The role of GBS in neonatal sepsis may be due to (i) it being one of the most prevalent colonizers of the birth canal and thus among the first bacteria to get into contact with the newborn (4, 5), (ii) GBS carrying highly invasive properties, and (iii) a particular neonatal immunopathology induced by GBS. In EOD, the size and deposition site, e.g., the lung, of the GBS inoculum may be decisive factors. However, it is unresolved why GBS establishes as a harmless mucocutaneous colonizer in approximately 10% of infants in the first weeks of life, and overcomes epithelial barriers and cellular innate immunity only in less than one in thousand infants to cause LOD. In other words, it remains a puzzle which specific factors at the level of mucosal immunity and the local microbiome allow GBS to leave its colonizing niche, thus facilitating invasion in the individual child.

At the beginning of life, the developmental lines of the microbiota and of the local cellular innate immunity have to run with substantial interdependence. Both areas are subject to factors *in cis* and *in trans*, i.e., specific bacteria are influenced by the microbiota and by host immunity, and host cells are modulated by other host and microbial cells (6). In order to guarantee long-term ecologic stability, adaptation on either side of the host–microbe interface is required, both at the population level and in the individual cell. The putative contribution of variations in specific innate immune genes to neonatal sepsis has recently been discussed (7). The authors suggested that affected children may suffer from yet to be identified minor primary immunodeficiency. This is a tempting hypothesis, given the enormous gain in knowledge on single gene alterations leading to susceptibility to a narrow spectrum of microorganisms. On the other hand, there is no indication for inheritance of a specific neonatal sepsis risk. Moreover, LOD typically remains the only “suspicious” episode in the individual infection biography. Finally, preterm birth is a well-recognized risk factor of GBS sepsis. In preterm infants, several factors impact on the individual codevelopment of microbiota and immunity, in particular cesarean section and formula feeding, which modify the microbiome (8, 9), and antibiotic usage, which affects both the microbiome and myeloid cell development (10, 11).

The hypothesis underlying this review holds that aberrations in the codevelopment of microbiota and host immunity, rather than genetic variations in immune genes alone, shape the individual risk for neonatal GBS sepsis, in particular LOD.

## GBS: Colonization and Virulence Factors

Neonatal GBS sepsis is a global problem with an overall incidence of around 0.5/1,000 live births. In contrast to the situation in Europe, American and African countries, GBS are reported to be a rare cause of neonatal colonization and sepsis in Southeast Asia (12, 13). However, the epidemiology in developing countries often suffers from constraints related to early deaths outside hospitals and low microbiological sensitivity of detection methods (13). In many, but not all Western European and North American countries, intrapartum antibiotic prophylaxis (IAP) has been associated with a decreased incidence of EOD while LOD rates remained unchanged (14–16). Notably, a substantial proportion of mothers whose infants developed EOD were tested negative before birth (1). It is unclear whether this phenomenon is due to false-negative test results or very recent GBS acquisition. Although, as outlined above, incidence and fatality rates are significantly higher in preterm than term infants (16–18), most cases occur in term infants (1) without clinical or laboratory evidence for immunodeficiency. LOD alone has an incidence of about 0.3–0.4 per 1,000 children and can develop randomly within the first 3 months after birth (19). It manifests more frequently as meningitis than EOD (17, 20). Conceptionally, these observations indicate that EOD and LOD originate from distinct biological processes or disturbances thereof.

Group B *streptococcus* is classified into 10 serotypes based on chemical structure and conformation of capsular polysaccharides. Serotyping relies on latex agglutination or multiplex PCR (21). In the past 30 years about 50% of the reported neonatal GBS sepsis cases worldwide were caused by serotype III strains (13). This indicates a considerable genetic homogeneity and stability in the pathogenic potential of GBS despite antibiotic selection pressure. Notably, Islam et al. did not detect any colonization by GBS of serotype III in their cohort of more than 600 infants in Bangladesh, while 6% of all infants were colonized by other serotypes, predominantly VII and Ia (22). It is very plausible yet uncertain that low circulation of highly invasive GBS strains underlies the low incidence of invasive neonatal GBS in several Asian countries (13).

In addition to the serotypes, GBS can be further classified by multilocus sequence typing, with more than 700 identified types (ST). The majority of human isolates belong to six clonal complexes (23, 24). EOD is significantly associated with serotype Ia strain ST-23 and closely related ST-24 as well as the ST-17 strain of serotype III (25, 26). LOD on the other hand is largely caused by ST-17 (20, 25). Moreover, ST-17 causes most cases of meningitis in EOD and LOD (27). In EOD, the distribution of invasive strains mainly corresponds to those colonizing the mothers (26). However, ST-17 shows an elevated disease-to-colonization ratio in EOD and LOD, i.e., it causes more cases of invasive disease than expected from its colonization rate of pregnant women (28–30). These observations, together with the characteristic expression of several virulence factors, have led to the term of a “hypervirulent” strain. Two of these factors, the hypervirulent GBS adhesin HvgA (27) and the serine-rich repeat glycoprotein Srr2 (31), are surface-anchored proteins which allow for adherence to epithelial cells and host plasma proteins. ST-17 strains also often carry the 2b pilus variant which contributes to invasion in mouse models (32, 33).

Most GBS strains produce surface-associated β-hemolysin which can damage membranes and promote barrier penetration (34). β-Hemolysin was found to be identical to the orange to red pigment of GBS, an ornithine rhamnolipid called granadaene (35). Both factors rely on the *cyl* operon which is controlled by the CovR/S two-component system. Strains mutated in CovR/S show hyperhemolysis and increased virulence (34, 35). For further detailed descriptions about GBS virulence factors, we refer to recent reviews (36, 37).

## Routes of Infection

In EOD, GBS is usually transmitted from the colonized maternal vaginal tract during birth to the infant. Aspiration of contaminated fluids allows for bacterial entry *via* the respiratory tract in many cases, resulting in sepsis or pneumonia during the first days of life (38). The route of infection in LOD is less well understood. The gastrointestinal tract is considered to be a natural reservoir for sepsis pathogens in neonates (39, 40). GBS shares this niche with *Escherichia coli*, the second typical organism in neonatal sepsis. Yet, the point of time when GBS establishes colonization is highly variable. 50–70% of colonized mothers transfer GBS to their offspring during delivery (41, 42) and 50% of infants which later developed LOD were colonized with GBS at birth (43). It remains unknown how many of these infants were stably colonized between the first contact with GBS and the disease onset. Unfortunately, large-scale and longitudinal colonization data of mother-infant pairs before and after disease onset, which would allow resolving this LOD puzzle, are not available. In a case series, Carl et al. found that 7 out of 11 children with LOD by GBS, *E. coli* or *Serratia marcescens* produced at least one stool with the matching organism before bloodstream infection (39). However, only two infants with GBS sepsis contributed to this study and they showed a GBS positive stool only briefly before sepsis, indicating recent colonization or overgrowth in the gastrointestinal tract. Another longitudinal case study on LOD also found that GBS occurred in the stool 2 days before sepsis onset (44). In contrast, it has been shown for other infections, e.g., enterococcal or staphylococcal bloodstream infections, that children often have a pathogen-dominated gut flora before disease onset (44, 45). Thus, it is conceivable that GBS exposure constitutes a particular LOD risk to infants who failed to firmly establish GBS colonization after birth (46). However, it seems important to note that stool samples do not always adequately mirror the actual intestinal community (47).

Meningitis caused by serotype III strains is often linked to high-level bacteremia. Factors that enable serotype III strains to survive in the blood stream, i.e., escape of adaptive and innate immune mechanisms, such as antibody or complement-mediated phagocytosis may be responsible for this effect (48). While the route of infection has not been resolved with certainty in infants, several studies showed bacterial dissemination to the blood and CNS after intraperitoneal (49), subcutaneous (50, 51) and intragastral (27, 52) inoculation of GBS serotype III in neonatal mice and rats. ST-17 is also specifically found in cases of GBS meningitis after 3 months of age (53), indicating that this clonal complex has an increased capability of overcoming colonization site barriers and blood borne immunity and of invading the CNS.

## The Neonatal Microbiome

The microbiome, defined as the microbial flora inhabiting the human body, constitutes an important factor in individual health and development. The composition of the microbiome is complex, distinct between individuals and subject to environmental changes and adaptation to host factors. Each body site contains a unique microbial community. Even within one niche such as the skin the composition varies depending on the exact location, i.e., the back skin shows a different microbial signature than the foot pad or the axillary vault (54). It seems self-evident that exposure to bacteria in the birth canal impacts on the colonizing flora in the infant. However, the fetus may be less sterile than thought, i.e., that the microbiome might develop already *in utero*. 16S rDNA sequencing of amniotic fluid, placenta samples and meconium revealed prenatal presence of bacteria with a predominance of *Escherichia* spp. (9, 55, 56). Of note, the *Streptococcus* genus was also detected in these samples, yet at very low abundance (56). Intrauterine colonization data have to be interpreted with some caution, since microbial viability is usually not confirmed and the risk of contamination is high in many of the investigated samples (57). Accordingly, the contribution of colonization *in utero* to microbiome development is still unclear, whereas that of colonization after rupture of fetal membranes is beyond doubt. As an example, vaginal delivery and cesarean section result in different bacterial communities on skin, nares, and gingiva (9). Yet, the impact of the delivery mode on the expansion and functional diversification after the first 6 weeks of life is surprisingly modest (9, 58). Instead, the infant’s microbiome follows a rather predictable successive colonization pattern and reaches a stable state resembling the adult microbiome already at 1–3 years of age (59–61). Oxygen abundance in the neonatal gut facilitates the colonization by facultative anaerobes, e.g., *Lactobacillus* and *Streptococcus* followed by *Enterobacteriaceae*. After oxygen is consumed and anaerobic conditions are established, obligate anaerobic species, e.g., *Bifidobacterium, Bacteroides*, and *Clostridium* spp. populate the intestine (62, 63). Administration of antibiotics, on the other hand, heavily affects the postnatal microbiome (8, 64, 65). Postnatal exposure to antibiotics alters the gut microbiome in the first 2–3 years of life by delaying microbiome development and altering phylogenetic diversity, e.g., affecting early colonization with *Lactospiraceae* spp. (8, 65). In addition, antibiotics reduce the stability of the microbiota composition as indicated by an increased variation between consecutive samples as compared to controls (65). Notably, very preterm infants with a gestational age of <33 weeks, who in many cases receive antibiotics within 24 h of birth, showed a 10-fold reduced bacterial diversity in comparison to term infants (66).

## GBS as Part of the Human Microbiome

*Streptococcus* is, together with *Lactobacillus, Staphylococcus*, and *Propionibacterium*, one of the most commonly found bacterial genera in the neonatal intestine and oral cavity (9). Streptococcal species account for up to 10% of total bacteria in fecal samples during the first months of life (67–69). In pregnant women, GBS colonization is found in up to 30% of rectovaginal samples (28, 70, 71) and stable colonization with the same clone for several years has been demonstrated (4, 70). Spread from the gastrointestinal tract to the genital tract is considered to be a probable colonization sequence for GBS (4). Since strains might be lost or reacquired in relatively short time periods (72, 73), GBS screening is recommended relatively late in pregnancy, i.e., between gestational weeks 35 and 37 (74).

Colonization by GBS is not exclusively confined to humans. Instead, GBS was first described in the 1880s as a cause of mastitis in goats and cows and it is a frequent commensal in seals and fish (75, 76). Although rare, invasive GBS disease can be a zoonotic disease as outbreaks in adults have been linked to raw fish consumption (77). Moreover, the hypervirulent ST-17 strain, which emerged 40 years ago, shares greater genetic similarity with bovine than with many human strains, indicating that it originated from a bovine lineage. Therefore, GBS may—under very specific conditions—cross species barriers (28, 78). However, since virulent strains in humans are distinct from those causing disease in animals (26, 75), person-to-person transmission plays the primary role in human GBS dissemination. Data on GBS spread are largely confined to mother-infant pairs. In contrast, the contribution of fecal-oral transmission by other family members than the mother to GBS colonization of the infant remains unclear. While strains are largely shared between sexual partners (79, 80), cohabitation appears to play a minor role in transmission (81).

Intrapartum antibiotic prophylaxis during delivery may transiently increase the GBS colonization risk of the infant yet probably does not affect the relative abundance of *Streptococcus* spp. in the stool beyond the first few weeks of life (72). While a number of studies longitudinally analyzed the development of the microbiome after birth on the level of phylum, class or order, studies on species or even genus level, e.g., with a specific focus on Group A *Streptococcus* (GAS) or GBS are rare and do not allow for robust statements on this level of resolution. Infants which were tested negative for GBS after IAP administration frequently acquire maternal GBS strains at later time points (82). Breast milk is hence a probable source of GBS in LOD. Several LOD case studies detected GBS in breast milk (46, 83). However, it is often unclear whether GBS in breast milk results from maternal colonization or infant oropharyngeal contamination. Mutated strains from infants which have been detected in the maternal breast milk (84) support the latter hypothesis. On the other hand, positive cultures of breast milk correspond to heavy colonization of the newborn (82), which is in turn a risk factor for LOD, especially in the case of mastitis (18). Bacterial expansion in breast milk and subsequent uptake by the infant may favor heavy colonization and LOD recurrences. Finally, nosocomial GBS transmission can occur in the case of children with invasive devices (82), indicating again that LOD can be a smear infection in some cases.

## Competing Microbes: GBS Needs to Find Its (Neonatal) Niche

Although GBS is the most prevalent streptococcal strain in neonatal sepsis, other streptococci, notably Groups A, D, and G, are isolated from blood cultures of newborns as well (22, 85, 86). Indeed, the connection of GBS and neonatal sepsis was only found in the 1960s and its predominance was established in the 1970s (24, 78). Prior to that, GAS and *Streptococcus pneumoniae* accounted for most neonatal sepsis cases (3, 87). As in other ecological niches, competition for nutrition and space occurs between bacterial species on colonized human body sites (88). Indeed, examples of mutual exclusion are found in the genus *Streptococcus*, e.g., in the case of *Streptococcus mutans*, the predominating cause of caries. The presence of other streptococcal species in the oral cavity, namely *Streptococcus sanguinis* and *Streptococcus oligofermentans*, is inversely correlated with the abundance of *S. mutans* which has been linked to the production of hydrogen peroxide *in vitro* (89, 90). Another example is the observation that *Corynebacterium* and *Dolosigranulum* in the upper respiratory tract are protective against colonization with *Streptococcus pneumonia*, which causes otitis media in infants after colonization of the airways (91). More importantly in the context of this review, growth of GBS is inhibited by *Streptococcus salivarius* both *in vitro* and in a vaginal colonization mouse model (92). Competitive growth was also shown for *Bifidobacterium* and GBS *in vitro* (93) and lactobacilli inhibited growth (94) and attachment of GBS to vaginal epithelial cells (95). In addition, *Lactobacillus reuteri* reduced vaginal colonization in a mouse model (96) and—importantly—as a probiotic in a placebo-controlled trial in pregnant women (97). These findings are in line with a very recent randomized, double-blind, placebo-controlled trial from Indian, where *Lactobacillus plantarum* plus fructooligosaccharide protected newborns from sepsis (98). In general, however, the presence of GBS appears not to be linked to an abnormal microbiome or a reduction of the predominant *Lactobacillus* genus in the vaginal tract of the mother (99–101). Interestingly, a small study found significant taxonomic differences in stools of 6-month infants, when mothers were GBS carriers, as compared to non-carriers (102). Yet, robust epidemiological evidence for a correlation of neonatal colonization with GBS and that of other specific intestinal commensals such as other streptococcal species is not existent.

Next to streptococci, staphylococci cause bacteremia and sepsis in newborns. Indeed, coagulase-negative staphylococci are the most common cause of nosocomial sepsis in newborns, yet do not play a role in healthy term infants. The generally more virulent *S. aureus* is isolated in variable frequency from neonatal blood cultures, but it is rarely found in cerebrospinal fluid (86). Furthermore, in view of the omnipresence of *S. aureus* as a colonizer in up to 50% of neonates, infants of this age group are not specifically susceptible to staphylococcal infections, unless they are subject to medical interventions such as indwelling catheters or surgery (85, 103). Hence, the contact with GBS and potentially other (beta-hemolytic) streptococci and the establishment of coexistence with these bacteria appears to impose a greater risk to the infant compared to other genii.

## The Impact of Antibiotic Pressure and Resistance on LOD

The majority of GBS strains isolated from humans are resistant to the antibiotic tetracycline. Indeed, the insertion of tetracycline resistance (TcR) elements, i.e., the ribosomal protection proteins Tet(M) and Tet(O), in few GBS clones led to their selection and expansion after the onset of extensive tetracycline usage since 1948 (24). These clones have since replaced a prior diverse GBS population, concurrent with the rise of GBS as major cause of neonatal sepsis. Notably, TcR elements are the most widely spread resistance genes in the human gut microbiota (104). Moreover, a subset of GBS strains, especially ST-1, carry genes which confer general resistance to macrolids and lincosamides, i.e., the methylases erm(B) and erm(TR) (24). Resistance rates to clindamycin (lincosamid) and erythromycin (macrolide) range up to 30 and 50%, respectively (30, 71, 105, 106). A rise of resistance to fluoroquinolones has been described in serotype V strains (105, 107). In addition, GBS with reduced penicillin susceptibility due to mutations in the penicillin-binding proteins are isolated with increasing frequencies in Japan (108, 109) and were also reported to occur spontaneously in an American patient after prolonged penicillin treatment (110). In this context, it seems likely that the frequent use of antibiotics other than tetracyclines may also lead to selection of hypervirulent strains. In the Netherlands, the incidence of EOD caused by ST-17 has significantly increased after implementation of a risk-based approach of antibiotic prophylaxis (15). ST-17 strains are also significantly more prevalent in women with IAP as compared to other strains (72). Thus, a relatively short course of intrapartum antibiotics, usually penicillin and ampicillin, may allow for seeding and expansion of hypervirulent GBS strains, which may not affect the majority of infants but propagate LOD development in few colonized individuals.

In addition, the capsular serotypes of GBS are not fixed but subject to frequent exchange by conjugative transfer between strains, explaining for the diversity of serotypes within clonal complexes. Lately, serotype IV has emerged as a causative agent of adult GBS disease in the US (106, 111). This seems important, as serotype IV is not included in the latest efforts in vaccine development to capsular antigens of GBS. Sequencing has revealed that a predominating serotype IV strain acquired large genomic fragments by horizontal gene transfer from the hypervirulent ST-17 and ST-23 strains (112). Additionally, ST-17 strains with capsular switching to serotype IV have been identified in several countries (29, 113, 114). Since maternal antibodies can impact on colonization with the antibody-specific GBS strains in mothers and early infants (115–117), it remains an open question whether targeting certain serotypes may eventually select for strains which have acquired novel capsule genes and allow for their expansion.

Interestingly, single-nucleotide polymorphisms (SNPs) in virulence-associated genes were detected in neonatal invasive GBS strains in comparison to the respective colonizing strains from the mothers, possibly contributing to the transition from a maternal commensal to a neonatal pathogen (84). This suggests that mutations are positively selected for in the neonatal environment. Moreover, mutations in the virulence regulator CovR/S leading to hyperhemolytic activity were found in invasive isolates of women in preterm labor (35). The acquisition of antibiotic resistance, serotype switching and SNPs can therefore lead to microevolution in the individual newborn, which may explain the pathogenicity of GBS in only a very small number of infants.

## The Role of Antibiotics and Dysbiosis in the Development of GBS Sepsis

The microbiota may have beneficial but also detrimental, acute, and chronic effects on infant health. Dysbiosis may predispose the neonatal intestine to inflammation (63) and facilitate the expansion of otherwise infrequent pathobionts (118, 119). Dysbiosis with lower bacterial diversity and decreased density of *Propionibacterium* spp. was found to precede the onset of necrotizing enterocolitis (NEC) (120, 121). Moreover, lactate-producing bacilli such as staphylococci and streptococci were reduced after birth in infants with NEC (68). Even though the increased prevalence of opportunistic pathogens such as uropathogenic *E. coli* (122) and Clostridium perfringens (68) has been linked to NEC, a common bacterial signature has not been found (121, 123). In addition, it is often unclear whether dysbiosis and the development of organ pathology are causally linked or whether they both depend on upstream disturbances, which may be diverse. Mai et al. found signs of dysbiosis in preterm infants already 2 weeks before onset of sepsis (124). Dysbiosis meant a delayed colonization with *Proteobacteria* and decreased density of *Bifidobacteria* spp. This observation receives support by the finding that *Bifidobacterium* spp. in the gut are protective for LOD (44), although the data on this issue are not fully consistent between studies (40). During sepsis, anaerobic *Bacteroides* and *Bifidobacterium* spp. were found to be decreased and aerobic *Enterobacteria* to be increased in affected infants as compared to non-septic twin controls (125). In view of these observations, a reduced intestinal *Bifidobacterium* density in infants whose mothers received IAP constitutes an important warning sign for the most careful usage of antibiotics in this sensitive period (93). In support of this notion, the risk for LOD caused by various pathogens including GBS in preterm infants is threefold higher after prolonged empirical antibiotic treatment (126). Antibiotics can affect the composition of the microbiome in many ways, including the depletion of competitive microbes, a delay in immune cell maturation (see below) and dysbiosis, all of which widen the niche for pathogenic bacteria.

## Cellular Innate Immunity and Resistance to GBS

Group B *streptococcus* is also recognized as an important health threat in immunocompromised adults, i.e., the elderly and patients with diabetes mellitus or HIV infections. Notably, the most common manifestations are skin/soft tissue infections and bacteremia (127–129), indicating that in these patients barrier immunity is important for the normal containment of GBS, similar to the situation in infants. The immaturity of the neonatal immune system in comparison to that of the adult was reviewed in detail elsewhere (130–132) and we will therefore focus on selected GBS-related aspects.

Neonatal rodents show exquisite sensitivity for GBS. Neonatal rats succumb to doses as low as 10 CFU intraperitoneally, while adult rats require approximately 6-log higher inoculums for a similar mortality rate (49) even if their body weight is taken into account (50). Neonatal mice, which normally die after i.p. infection within 48 h, were protected by transfer of specific antiserum to the pregnant dam before delivery (133). This experimental data is in line with the protective role of maternal GBS antibodies in the protection from GBS EOD, which is the basis for the development of a maternal vaccine (36, 134). In contrast, the role of maternal antibodies in the prevention LOD development is less clear. Recently, it has been inferred that high antibody levels also prevent GBS colonization (42, 116, 117). Women with high serotype-specific titers had a significantly lower risk of rectovaginal colonization with the respective GBS strains (42). However, GBS antibody levels do not inversely correlate with the sepsis risk *per se*. Thus, it remains puzzling why only very few of the GBS exposed and/or colonized infants with low antibody levels develop LOD.

In the innate arm of the immune system, the family of Toll-like receptors (TLRs) is essential for the defense against invasive streptococcal infections. Children with genetic deficiency in MyD88, an essential adaptor for all TLRs but TLR3, or IRAK4, a kinase downstream of MyD88, have an approximately 50% risk of dying from invasive bacterial infections in the first 8 years of life. In most cases, streptococci are the causative organisms (135, 136). Furthermore, roughly one third of the affected children suffer from a sepsis episode in the first 3 months of life. Thus, the risk for early and late neonatal sepsis is approximately 1,000-fold higher in these infants than in newborn infants overall. It seems noteworthy that most isolates are either pneumococci or GAS, whereas only few cases of late neonatal sepsis and meningitis caused by GBS have been reported so far (135, 137). Whether this predominance of other streptococcal species is due to an altered microbiome in MyD88- and IRAK4-deficient individuals has not been explored so far. In mice with MyD88 deficiency, a gross deviation in microbiome composition cannot be observed (138, 139), although a generally increased risk for the invasion and dissemination of intestinal commensals was observed (140). Moreover, MyD88-deficient neonatal mice have not been studied in this context. The already exceptional susceptibility of neonatal mice for local GBS infections, with a 100,000-fold decreased LD90 (cfu/g bw) in 2-day-old mice as compared to adult mice, is further significantly increased in MyD88 deficiency (141, 142).

**Figure 1:**
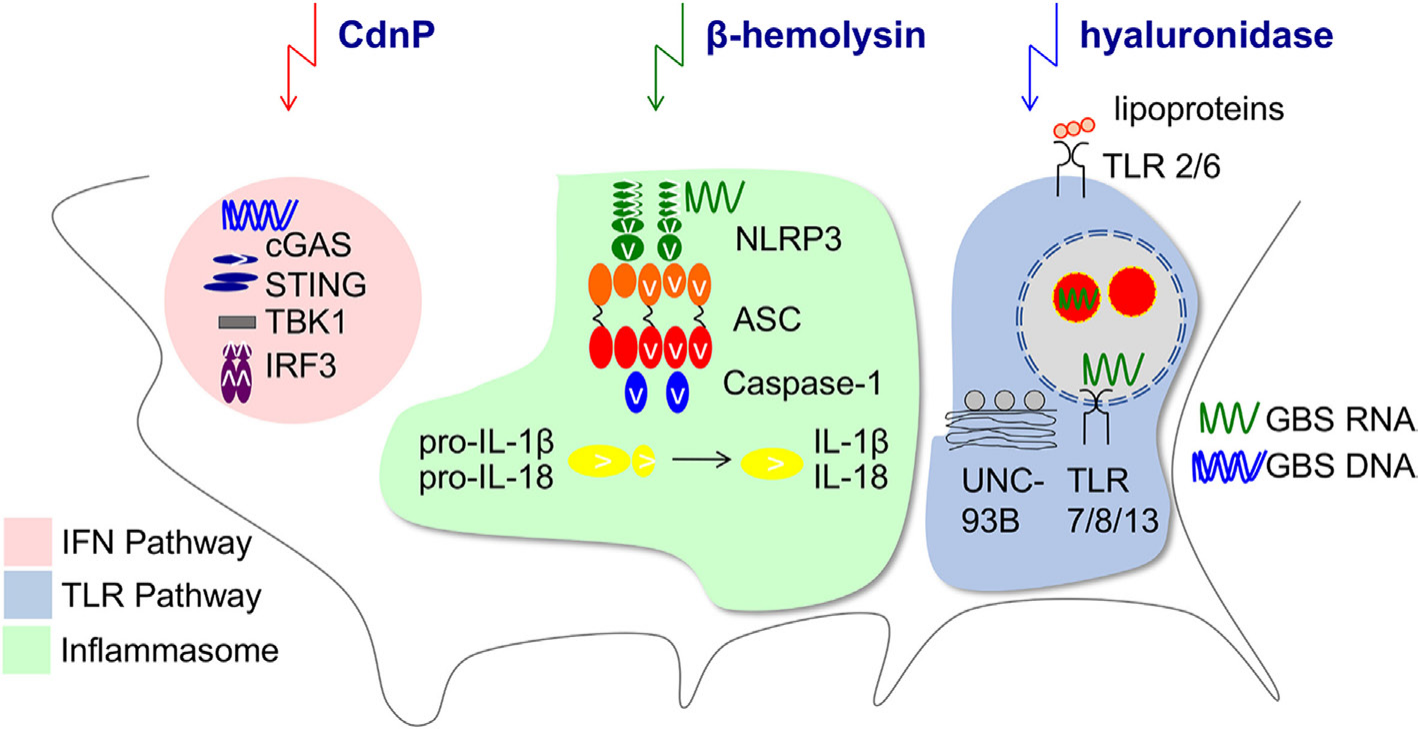
Innate immune pathways manipulated by Group B *Streptococcus*. Depicted is the impact of GBS on type I interferons (IFN) (153, 155), Toll-like receptor (TLR) (10, 149), and inflammasome (151) pathways by secreted bacterial factors. The ectonucleotidase CdnP hydrolyzes bacterial cyclic dinucleotides which otherwise activate STING and IFN-β production (154). Hemolysin contributes as second signal to the NLRP3 inflammasome activation (152). The GBS hyaluronidase can degrade pro-inflammatory hyaluronan polymers during tissue injury which normally bind to TLR2 and the resulting fragments block TLR2 signaling in the host (157).

Within the MyD88-dependent TLR family, TLR2 activation by GBS lipoproteins (143, 144) and endosomal TLR-activation by single-stranded RNA are equally important. TLR13 is a common receptor of 16S rRNA from Gram-positive bacteria including GBS in mice (10, 145), whereas TLR8 is the incomplete analog in humans (146–148). TLR recognition by myeloid cells is highly site-specific, i.e., RNA sensing and TLR13 are crucial for recognition of GBS by resident mouse macrophages but not circulating blood monocytes (142). Interestingly, recognition of GBS and Gram-positive bacteria appears to rely more on endosomal TLRs than recognition of Gram-negative bacteria (149). This seems intriguing in the context of human neonatal mononuclear cells, which are particularly responsive to TLR8 ligands (150). Accordingly, recognition of bacterial RNA by TLRs is not only particularly important at the beginning of life, but may result in distinct immune activation patterns induced by *Streptococcaceae* and *Enterobacteriaceae*. It remains an appealing yet unproven hypothesis that TLR8-dependent immunopathology contributes to myeloid cell-mediated disturbance of mucocutaneous barrier integrity. In addition, TLR8 and 13 do not hold exclusive roles in the recognition of GBS RNA or nucleic acids in general. First, the NLRP3 inflammasome mediates GBS-induced formation of IL-1β and IL-18 in macrophages *via* recognition of ssRNA (151, 152). NLRP3 activation requires the induction of potassium efflux by a rhamnolipid of GBS, which also mediates cytolysis (35). Proper inflammasome activation is essential for the neonatal resistance against GBS (151). Next, GBS DNA engages the cytosolic signaling of cGAS and STING which leads to interferon (IFN)-β production and contributes to GBS immunity (153, 154). In addition, conventional dendritic cells secret type I IFNs in response to endosomal GBS RNA interacting with TLR7 (155). GBS may subvert nucleotide sensing *via* expression of ectonucleotidases (154, 156) (Figure 1). Similarly, the GBS hyaluronidase HylB blocks cellular activation by degrading host hyaluronic acid into fragments which bind and inhibit TLR2 (157). HylB was shown to promote vaginal colonization and ascending infections in mice (157, 158). How these enzymes impact on the sensing of colonizing GBS and of competing bacteria in neonates is currently unclear. It furthermore remains to be determined how the relatively increased TRIF-dependent pathway in neonates impacts on barrier defense against GBS (159). Any effect can be assumed to be indirect, since TRIF is redundant in GBS-mediated activation of phagocytes, although a role as a signaling intermediate in other (immune) cells cannot be excluded (149, 160).

Understanding the distinct TLR, inflammasome and cGAS engagement in the monocyte-macrophage lineage by GBS is of utmost importance, since macrophages are the dominant resident immune cells at mucocutaneous barriers, i.e., the dermis and the gut. They are crucially involved in barrier maintenance (161, 162), both by executing direct antimicrobial actions and by cytokine and chemokine dependent recruitment and activation of other immune cells. Development of the neonatal macrophage compartment is particularly well understood in the neonatal intestine, where the population of embryonic macrophages is replaced by monocyte-derived macrophages starting at weaning (163). It is tempting to speculate that macrophage maturation in the lamina propria directly impacts on the macrophage-driven recognition and elimination of invading GBS. Another TLR-based mechanism promoting susceptibility to GBS is the increased production of anti-inflammatory cytokines. Enhanced IL-10 concentrations in serum and cord blood are correlated with mortality in septic infants (164). Moreover, IL-10 has a major impact on intestinal barrier immunity, both in humans and mice. Yet, whereas too little IL-10 leads to spontaneous inflammation and colitis, increased IL-10 production impairs neutrophil recruitment into infected organs and thus decreases GBS clearance (164, 165). How increased IL-10 formation impacts on keeping GBS in a colonization—as opposed to an invasion—state is currently not known.

## Impact of the Microbiome on the Developing Immunity

**Figure 2:**
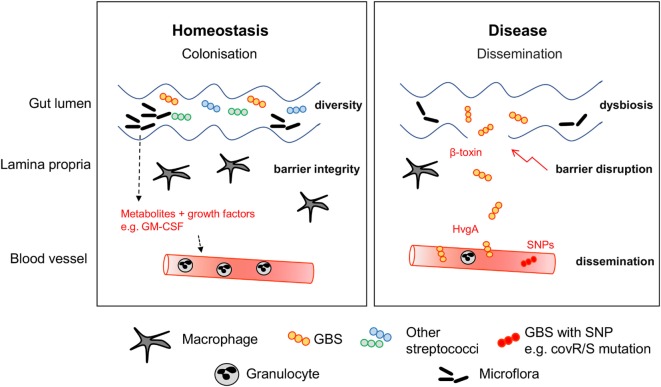
Stabilization of the mucocutaneous niche. During homeostasis, GBS colonizes the intestine of healthy infants. Macrophages and other immune cells guarantee barrier integrity by surveillance. Other commensal bacteria including streptococcal species form the niche. Disease can be preceded by multiple factors leading to dysbiosis, expansion of GBS and barrier disruption. Expression of virulence factors such as HvgA and β-toxin facilitate adhesion to epithelial cells and barrier disruption. Dissemination is often concurrent with mutations of the CovR/S virulence repressor.

Numerous studies were initiated to understand the impact of the colonizing flora on the function of intestinal cells in general and the immune system in general. Research is usually based on germ-free mice and antibiotic treatments in order to understand the consequences of a reduction or absence of microorganisms. Evidence for immunological consequences of alterations in the microbiome was even found in cells very distant to the gastrointestinal tract such as brain microglia (166). In a highly interesting mouse study, exposure of the pregnant dam to antibiotics not only led to neutropenia in newborn mice, but subsequently increased the susceptibility to Gram-negative sepsis (10). A reduction in *Gammaproteobacteria* may mediate these effects, since their effector LPS induces granulocyte colony-stimulating factor production and consequently granulopoiesis. Recently, Josefsdottir et al. suggested that the microbiota is the cause of neutropenia and general depletion of hematopoietic stem cells across multiple lineages in antibiotic-treated mice (11). The phenotype could be partially rescued by fecal transfer. This experimental data is in line with the observation that administration of ceftalorine and β-lactam antibiotics can lead to neutropenia in patients (167, 168). Consequently, antibiotics appear to indirectly impact on the maturation of the immune response (169) and the resistance against neonatal sepsis pathogens. An overall smaller granulocyte pool in neonates (132) may further propagate the negative effects of antibiotics. Therefore, it seems that the immaturity of neonatal blood cells, including phagocytes and adaptive immune cells, might restrict the ability to fight off pathogens. Hence, in the stochastic event of pathogen invasion through the muco-cutaneous barrier, which may be potently responded to by the adult immune system, neonatal immunity may be overwhelmed, resulting in bacterial spread and sepsis (Figure 2). It remains incompletely understood whether the protection in the adult usually involves the resident immune cells at mucocutaneous sites, e.g., the lamina propria in the gut or the dermis in the skin, or whether circulating leukocytes are necessary for efficient barrier defense.

## Conclusion

The challenge to understand and ultimately prevent neonatal GBS sepsis comprises (i) the control of GBS transmission during and immediately after birth leading to EOD and (ii) the subsequent control of GBS as a mucocutaneous colonizer, when failure results in LOD. Whereas high maternal antibody titers, as induced by GBS vaccines, and IAP are established strategies to prevent EOD, similar strategies with proven efficacy for LOD reduction are missing. Based on experimental and observational evidence, it seems worth considering—and thus requires careful studies—whether antibiotic pressure during primary colonization of the intestine facilitates dysbiosis on the strain level and transient immunodeficiency in the individual child. Furthermore, capsular polysaccharide based vaccines may select for serotype-switched virulent strains as observed with ST-17 and allow for the expansion of other β-hemolytic streptococci than GBS.

The vast recent gain in knowledge on the coevolution of microbiome and cellular barrier defense make the design of novel approaches for neonatal sepsis prevention conceivable, although much preclinical work remains to be done first. Examples are designer probiotics, containing—among others—strains which occupy the streptococcal niche without risk of invasion. Immunomodulators that accelerate the maturation of the phagocyte population resident at mucocutaneous sites may be another strategy that holds potential. Yet, the variable conditions and demands at the beginning of life, e.g., that of very preterm infants or those requiring antibiotic therapy early on, make one-fits-all solutions to the neonatal sepsis conundrum unlikely and rather ask for individualized approaches.

## Author Contributions

JK and PH wrote and edited the manuscript.

## Conflict of Interest Statement

The authors declare that the research was conducted in the absence of any commercial or financial relationships that could be construed as a potential conflict of interest.
